# Supplementary material for: Pupillometry as a reliable metric of auditory detection and discrimination across diverse stimulus paradigms in animal models
Source: Sci Rep. 2021 Feb 4;11:3108. doi: 10.1038/s41598-021-82340-y (PMC7862232; doi:10.1038/s41598-021-82340-y)
Supplement: Supplementary file 1 — Supplementary Information 1. [file 41598_2021_82340_MOESM1_ESM.pdf]

Supplementary Information

Pupillometry as a reliable metric of auditory detection and discrimination across diverse stimulus paradigms in animal models

Pilar Montes-Lourido, Manaswini Kar, Isha Kumbam, Srivatsun Sadagopan

University of Pittsburgh

Supplementary Information contains:

1 Supplementary Video (MP4)

1 PDF file with:

- Legend for Supplementary Video
- Supplementary Figs. 1 – 3 and Legends
- Supplementary Discussion
- 11 Supplementary Tables containing exact p-values for all statistical tests used in the main manuscript and supplementary materials.

Legend for Supplementary Video 1: Pupil diameter changes in an auditory oddball task.
Top panel: Video of GP eye under infrared illumination (2x speed).
Middle panel: White line corresponds to the pupil diameter trace. X-axis numbers are time in seconds. Gray bars correspond to white noise standards. Red bar corresponds to the deviant stimulus (in this case, a clean GP call).
Bottom panel: Voltage output of the piezoelectric sensor. Slower deflections correspond to respiration, rapid deflections in the later part of the trace corresponds to postural shifts made by the animal. Yellow line is time marker.

(continued on next page)

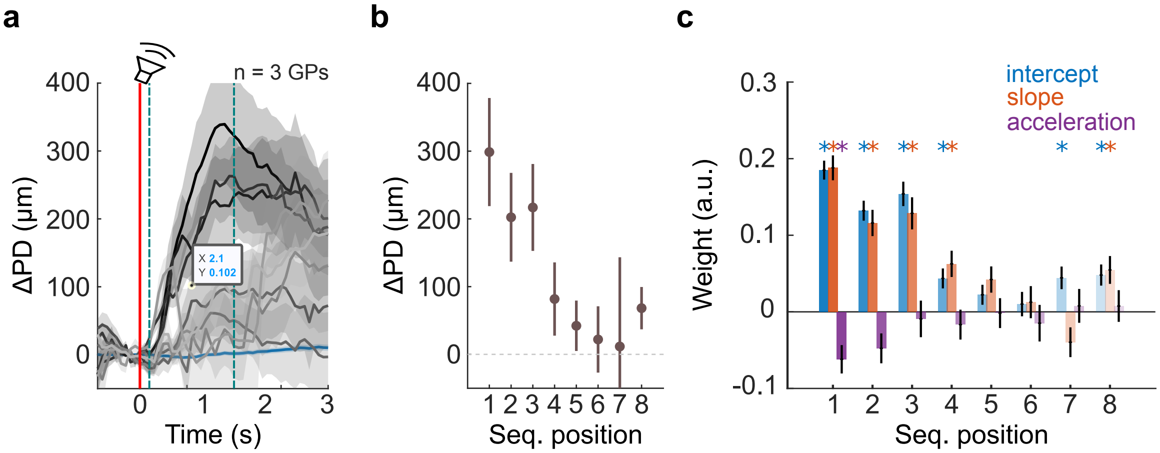

Supplementary Figure 1: Adaptation of pupil responses. (a) Pupil dilation in response to white noise bursts presented sporadically (~ once every 1.5 minutes). Darkest line and shading correspond to first noise burst, lightest line and shading to the last (eighth) burst. Red line corresponds to burst onset, teal dashed lines correspond to growth curve analysis (GCA) window. **(b)** Change in pupil diameter as a function of noise burst sequence position. Discs and error bars correspond to mean ± 1 s.e.m. **(c)** GCA weight estimates. Colors correspond to intercept (blue), slope (red) and acceleration (purple) terms of the model used to fit the rising phase of the pupil traces. Asterisks denote statistically significant regression weights ($p < 0.01$; see Supplementary Table 11 for exact p-values.)

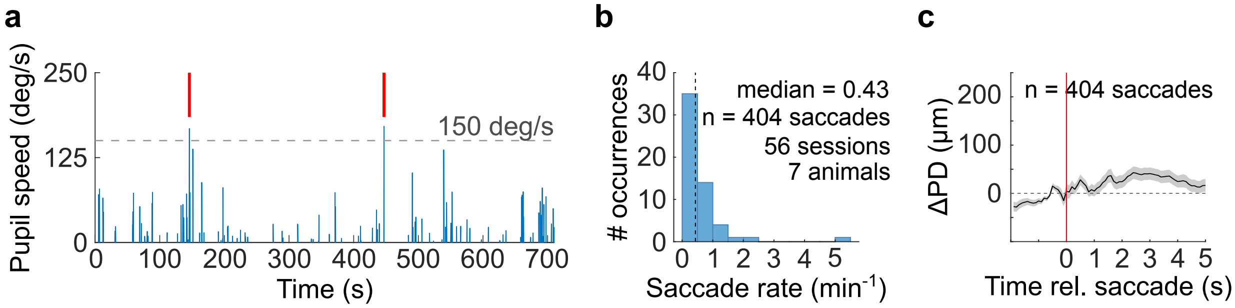

Supplementary Figure 2: Saccades are rare and minimally affect pupil diameter. (a) Pupil speed trace from one session of the harmonic tone discrimination paradigm. Red lines denote detected saccades, defined as pupil speed exceeding 150 deg/s. **(b)** Distribution of saccade rates measured in 56 sessions (across 7 animals) of the harmonic tone discrimination paradigm. We observed a median of 0.43 saccades per minute. **(c)** Mean pupil diameter change associated with a detected saccade event. Red line corresponds to saccade time. Line and shading correspond to mean ± 1 s.e.m.

Supplementary Figure 3: Spectrograms of guinea pig vocalizations used in pupillometry experiments. **(A)** Chut vocalizations (top) and Purr vocalizations (bottom) used in the experiments described in Figs. 4 – 6. **(B)** Chuts (top) and Purrs (bottom) with white noise added to result in a final signal-to-noise ratio of -3 dB SNR.

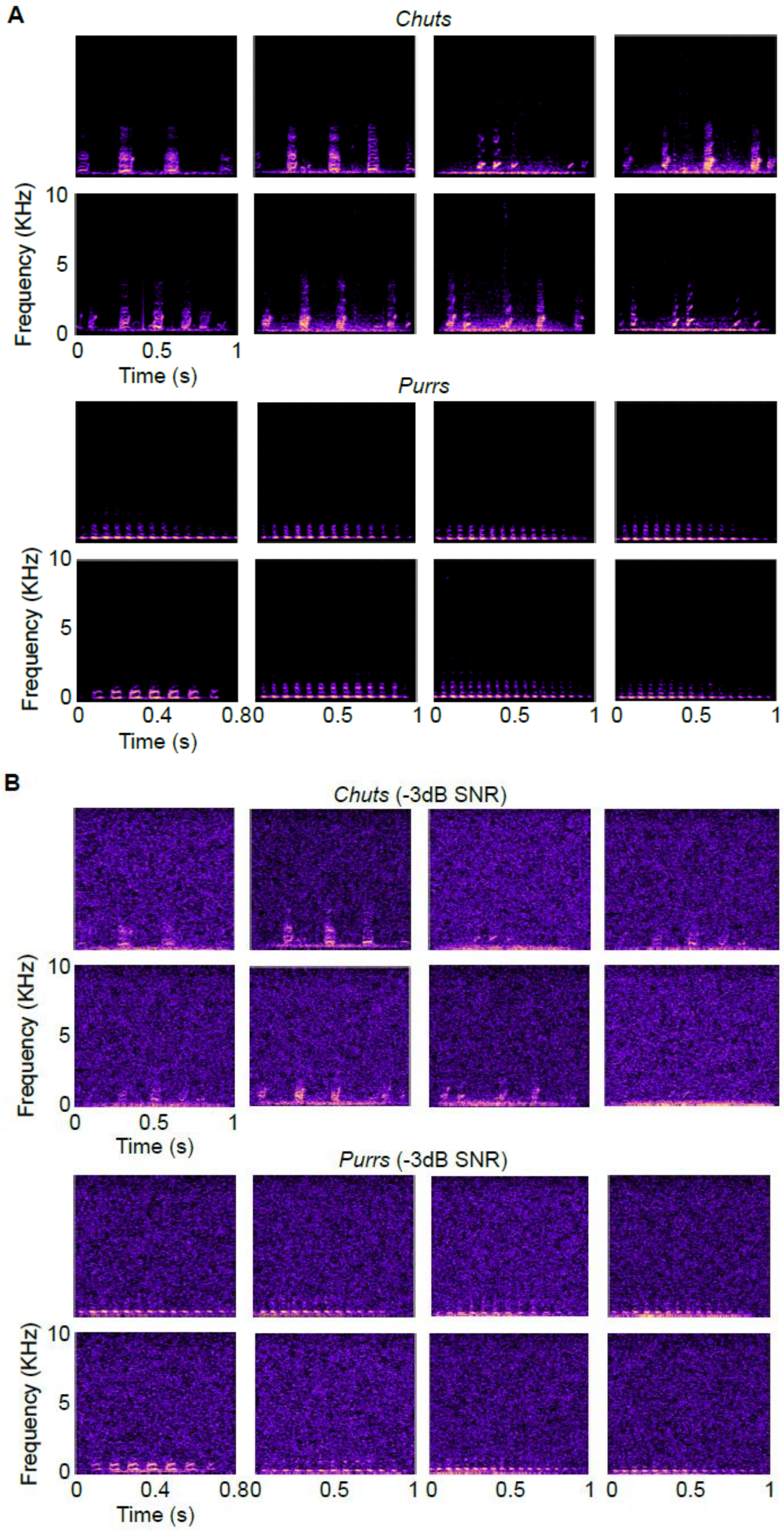

Supplementary Discussion

A minor methodological disadvantage is that in our experiments, animals were first surgically implanted with a headpost for head-fixation. Requiring head-posted animals potentially limits the scalability of using pupillometry for high-throughput evaluation of large experimental groups. While pupil tracking is possible in freely-moving animals, these systems also require some form of head-anchoring [1,2], and because pupil dilation accompanies animal motion [3,4], a free-moving preparation is a sub-optimal solution. Headpost-free restraints are possible – for example, for functional magnetic resonance imaging in small animals, 3d-printed ‘helmets’ have been used for headpost-free head fixation [5]. We believe that pupillometry with non-invasive restraint in non-headposted animals, using recent advances in markerless pose-estimation methods [6,7] to accurately track PD despite some head motion, is an easily achievable solution for acquiring high-throughput and high-quality pupillometry data.

1. Shepherd, S. V. & Platt, M. L. Spontaneous social orienting and gaze following in ringtailed lemurs (Lemur catta). *Anim. Cogn.* 11, 13–20 (2008).
2. Meyer, A. F., Poort, J., O’Keefe, J., Sahani, M. & Linden, J. F. A head-mounted camera system integrates detailed behavioral monitoring with multichannel electrophysiology in freely moving mice. *Neuron* 100, 46-60.e7 (2018).
3. McGinley, M. J., David, S. V. & McCormick, D. A. Cortical membrane potential signature of optimal states for sensory signal detection. Neuron 87, 179–192 (2015).
4. Vinck, M., Batista-Brito, R., Knoblich, U. & Cardin, J. A. Arousal and locomotion make distinct contributions to cortical activity patterns and visual encoding. Neuron 86, 740–754 (2015).
5. Silva, A. C. *et al.* Longitudinal Functional Magnetic Resonance Imaging in Animal Models. *Methods Mol. Biol.* 711, 281–302 (2011).
6. Mathis, A. *et al.* DeepLabCut: markerless pose estimation of user-defined body parts with deep learning. *Nat. Neurosci.* 21, 1281–1289 (2018).
7. Nath, T. *et al.* Using DeepLabCut for 3D markerless pose estimation across species and behaviors. *Nat. Protoc.* 14, 2152–2176 (2019).

Supplementary Table 1: Exact p-values for statistical analysis in Fig. 3E.

<u>Tests:</u> ANOVA ($F = 107.98$, $dF = 8$, $p = 1.63 \times 10^{-180}$), followed by Bonferroni-corrected post-hoc tests.

**(Baseline vs.) ΔF**	**p-val.**
0.25	0.072
0.50	1
0.75	6.2×10^{-7}
1.00	3.1×10^{-11}
1.25	1.4×10^{-31}
1.50	4.1×10^{-11}
1.75	2.8×10^{-74}
2.00	3.2×10^{-54}

Supplementary Table 2: Exact p-values for statistical analysis in Fig. 3H.

Tests: Linear hypothesis tests for model coefficients with Satterthwaite's correction for large degrees of freedom. For brevity, only significant p-values < 0.01, marked with asterisks in Fig. 3H, are shown.

ΔF (semitones)	Intercept p-val.	Time1 p-val.	Time2 p-val.
0.75	9.2×10^{-8}	0.0025	
1.00	3.5×10^{-8}	0.0057	
1.25	4.6 x 10-^{24}	4.8×10^{-8}	
1.50	3.7×10^{-11}		
1.75	6.0×10^{-43}	1.0×10^{-23}	5.1×10^{-5}
2.00	1.0×10^{-35}	3.6×10^{-16}	6.7×10^{-4}

Supplementary Table 3: Exact p-values for statistical analysis in Fig. 4F (*top*) *Chut* calls-in-noise.

Tests: Linear hypothesis tests for model coefficients with Satterthwaite's correction for large degrees of freedom. For brevity, only significant p-values < 0.01, marked with asterisks, are shown.

SNR (dB)	Intercept p-val.	Time1 p-val.
-4.5	1.6×10^{-7}	0.0019
-1.5	1.3×10^{-5}	1.1×10^{-4}
1.5	6.0×10^{-14}	7.9×10^{-12}
3.0	6.7×10^{-13}	2.0×10^{-6}
6.0	7.4×10^{-34}	1.5×10^{-16}
20.0	1.2×10^{-38}	3.6×10^{-25}

Supplementary Table 4: Exact p-values for statistical analysis in Fig. 4F (*mid*) *Purr* calls-in-noise.

Tests: Linear hypothesis tests for model coefficients with Satterthwaite's correction for large degrees of freedom. For brevity, only significant p-values < 0.01, marked with asterisks in Fig. 4F, are shown.

SNR (dB)	Intercept p-val.	Time1 p-val.	Time2 p-val.
-3.0	3.8×10^{-9}	0.0017	
0.0	2.6×10^{-12}	1.8×10^{-11}	
1.5	1.2×10^{-10}	2.3×10^{-9}	
3.0	0.0016		
20.0	3.3×10^{-80}	5.3×10^{-46}	4.8×10^{-6}

Supplementary Table 5: Exact p-values for statistical analysis in Fig. 4F (*bottom*) harm. tone-in-noise.

Tests: Linear hypothesis tests for model coefficients with Satterthwaite's correction for large degrees of freedom. For brevity, only significant p-values < 0.01, marked with asterisks in Fig. 4F, are shown.

**SNR (dB)**	**Intercept p-val.**	**Time1 p-val.**
-4.5	3.0×10^{-4}	0.0053
-3.0	1.0×10^{-5}	
0.0	2.4×10^{-4}	0.0024
1.5	0.0034	
3.0	1.8×10^{-26}	2.4×10^{-6}
6.0	6.3×10^{-12}	3.2×10^{-9}
20.0	4.0×10^{-7}	1.1×10^{-4}

Supplementary Table 6: Exact p-values for statistical analysis in Fig. 5C (*top*), call-in-noise detection for Days 1-4 of air puff conditioning.

Tests: Linear hypothesis tests for model coefficients with Satterthwaite's correction for large degrees of freedom. For brevity, only significant p-values < 0.01, marked with asterisks in Fig. 5C, are shown.

**SNR (dB)**	**Intercept p-val.**	**Time1 p-val.**	**Time2 p-val.**
1.5	2.5×10^{-4}		0.0039
3	1.5×10^{-14}	1.9×10^{-4}	
20	1.2×10^{-22}	6.8×10^{-15}	0.0011

Supplementary Table 7: Exact p-values for statistical analysis in Fig. 5C (*bottom*), call-in-noise detection for Days 8-10 of air puff conditioning.

Tests: Linear hypothesis tests for model coefficients with Satterthwaite's correction for large degrees of freedom. For brevity, only significant p-values < 0.01, marked with asterisks in Fig. 5C, are shown.

**SNR (dB)**	**Intercept p-val.**	**Time1 p-val.**	**Time2 p-val.**
-3	2.1×10^{-6}	8.7×10^{-5}	
-1.5	0.0022		
1.5	2.4×10^{-6}		
6	7.9×10^{-9}	0.0022	
20	3.9×10^{-43}	9.9×10^{-12}	0.0083

Supplementary Table 8: Exact p-values for statistical analysis in Fig. 6F, call categorization-in-noise without air puff conditioning.

Tests: Linear hypothesis tests for model coefficients with Satterthwaite's correction for large degrees of freedom. For brevity, only significant p-values < 0.01, marked with asterisks in Fig. 6F, are shown.

SNR (dB)	Intercept p-val.	Time1 p-val.	Time2 p-val.
3	2.3×10^{-4}	0.0064	
15	1.7×10^{-23}	7.3×10^{-10}	9.1×10^{-5}

Supplementary Table 9: Exact p-values for statistical analysis in Fig. 6G, call categorization-in-noise with air puff conditioning.

Tests: Linear hypothesis tests for model coefficients with Satterthwaite's correction for large degrees of freedom. For brevity, only significant p-values < 0.01, marked with asterisks in Fig. 6G, are shown.

SNR (dB)	Intercept p-val.	Time1 p-val.	Time2 p-val.
-3	1.3×10^{-4}		
-1.5	1.0×10^{-119}	1.1×10^{-71}	0.0024
0	1.7×10^{-97}	1.4×10^{-58}	0.0012
3	5.4×10^{-83}	1.4×10^{-35}	5.0×10^{-6}
4.5	7.4×10^{-47}	8.4×10^{-42}	0.0039
15	3.4×10^{-140}	5.7×10^{-96}	3.1×10^{-12}

Supplementary Table 10: Exact p-values for statistical analysis in Fig. 7E, figure-ground segregation.

Tests: Linear hypothesis tests for model coefficients with Satterthwaite's correction for large degrees of freedom. For brevity, only significant p-values < 0.01, marked with asterisks in Fig. 7E, are shown.

Coherence	Intercept p-val.	Time1 p-val.	Time2 p-val.
2	7.9×10^{-4}		
4	7.7×10^{-5}		
6		2.2×10^{-4}	
8	5.6×10^{-9}	7.9×10^{-4}	
10	3.3×10^{-13}	1.9×10^{-9}	0.0018

Supplementary Table 11: Exact p-values for statistical analysis in SFig. 1, adaptation.

Tests: Linear hypothesis tests for model coefficients with Satterthwaite's correction for large degrees of freedom. For brevity, only significant p-values < 0.01, marked with asterisks in SFig. 1, are shown.

**Seq. pos.**	**Intercept p-val.**	**Time1 p-val.**	**Time2 p-val.**
1	1.5×10^{-50}	6.6×10^{-31}	8.2×10^{-4}
2	3.8×10^{-24}	1.3×10^{-11}	
3	4.5×10^{-22}	8.5×10^{-10}	
4	8.0×10^{-4}	2.5×10^{-4}	
5			
6			
7	2.6×10^{-3}		
8	5.0×10^{-4}	2.6×10^{-3}	0.0018
